# Relationship between psychopathic traits and ability emotional intelligence in a sample of incarcerated males

**DOI:** 10.1192/j.eurpsy.2022.944

**Published:** 2022-09-01

**Authors:** R. Gómez-Leal, A. Megías-Robles, M.T. Sánchez-López, P. Fernández-Berrocal

**Affiliations:** Faculty of Psychology. University of Málaga, Department Of Basic Psychology, Málaga, Spain

**Keywords:** MSCEIT, Emotional Intelligence, Psychopathy, incarcerated population

## Abstract

**Introduction:**

The study of psychopathic traits has increased in recent years, given the impact that these traits have on our society.

**Objectives:**

This study aimed to evaluate the relationship between psychopathy traits and ability emotional intelligence by examining the sub-dimensions of both constructs in a sample of incarcerated males.

**Methods:**

A total of sixty-three incarcerated adult males (M_age_ = 37.51) were assessed for psychopathy traits and emotional intelligence levels through the 34-item Self-Report Psychopathy Scale-III (SRP-III) and the Mayer-Salovey-Caruso Emotional Intelligence Test (MSCEIT) respectively.

**Results:**

The results revealed that the incarcerated population is characterized by low EI and high psychopathic traits (explained by the scores obtained on the criminal tendencies sub-dimension). Moreover, participants scoring lower in ability EI were more likely to score higher on the callous affect sub-dimension of psychopathy. We also observed an indirect negative effect of ability EI on erratic lifestyle, criminal tendencies and interpersonal manipulation sub-dimensions through the mediating role of callous affect.

**Conclusions:**

These findings offer a better understanding of the relationship between psychopathy traits and ability emotional intelligence and provide empirical support for the need to implement intervention programs in penitentiary centers based on EI training, which could help to reduce antisocial and disruptive behaviours and facilitate future reintegration into society.

**Disclosure:**

No significant relationships.

